# Self medication practice and associated factors among students of Asmara College of Health Sciences, Eritrea: a cross sectional study

**DOI:** 10.1186/s40545-019-0165-2

**Published:** 2019-02-19

**Authors:** Zenawi Zeramariam Araia, Nahom Kiros Gebregziabher, Araia Berhane Mesfun

**Affiliations:** 1 National TB and Leprosy Control Program, Communicable Disease Control Division., Ministry of Health, Asmara, Eritrea; 2 School of Public Health, Asmara College of Health Sciences, Asmara, Eritrea; 3 Communicable Diseases Control Division, Ministry of Health, Asmara, Eritrea

**Keywords:** Self-medication, Adverse drug reaction, Health sciences, Developing countries, Eritrea

## Abstract

**Background:**

Self-medication is a common practice globally and the resulting irrational drug use is raising concerns. Up-to-date there is no systematic study conducted on self medication practice among students or the general community in Eritrea. The present study aimed to determine the prevalence of self-medication practice and its influencing factors among students of Asmara College of Health Science.

**Methods:**

A cross sectional study was conducted from 21st May -15th June 2018. Data on self medication practice and its associated factors was collected using a self-administered questionnaire. Data analysis was done using SPSS − 23 and explained with descriptive and inferential statistics.

**Results:**

A total of 313 students responded to the questionnaire with a response rate of 93.6%. The overall prevalence of self-medication practice was 79.2%. Headache and fever were reported as the most common complaint related to self-medication practice. Among the reasons for self medication practice, prior experience was the most frequently reported. Analgesics, antipyretic and antibacterial were the leading class of medicine used in self-medication practice while adverse drug reactions were reported by 9.2% of respondents. In this study, sex, income, and school of study were found to be the independent predictors for self-medication practice.

**Conclusion:**

National guideline on medicine access should be developed and strong measures should be implemented to halt the selling of medications without a proper prescription. In addition, students should be educated on the consequences of self-medication practices.

## Background

Self-care is practice or action taken by people for themselves in order to have and maintain health, avoid and protect from diseases. Self-medication is considered as one component of self-care [1]. According to WHO’s definition, “self-medication involves the use of medicinal products by the consumer to treat self-diagnosed disorders or symptoms, or the intermittent or continued use of medication prescribed by a physician for chronic or recurrent diseases or symptoms” [2].

A continuous worldwide increase in self-medication has been triggered by economic, political and cultural factors and the practice is becoming a major public health problem [3]. However, there is a difference in the prevalence of self-medication practices among developing and developed countries in relation to the variations in cultural and socioeconomic factors, dissimilarities in health care systems such as compensation rules, access to health care, and medicine dispensing policies [4]. In economically deprived countries, most events of illness are treated by self-medication, imposing much public and professional concerns about the irrational use of medicines [5]. A relatively higher percentage of medicines were being dispensed without a medical prescription or proper monitoring and this was attributed to a shortage of availability of healthcare service or health care service with trained health care workers is somewhat expensive. Consequently, self medication is becoming a noticeable option of health care services. Self medication can facilitate access to medicine and reduce healthcare costs [6, 7]. However, there are major problems associated with self-medication practice such as wastage of resources, increased resistances of pathogens and serious health risks like adverse drug reactions and prolonged suffering [8].

Numerous studies were conducted in different countries that investigated self medication practice among a different group of the population. According to the results of these previous studies complaints like a headache, fever, abdominal discomfort, sore throat, cramps and disease such as respiratory infections, malaria, pneumonia, eye infections, urinary tract infection, cold and gastrointestinal disorders were reported [4, 5, 9–12]. Similarly, numerous reasons were reported behind the practices of self-medication such as mildness of the disease, sufficient pharmacological knowledge, to save time, to avoid long waiting to see a doctor, suggestions of friends, inexpensiveness of the practice and previous experience [5, 9, 11, 13]. Analgesics, anti-pyretic, antibiotics, anti-acids, anti-microbial, anti-malarial, antihelmintiasis, antitussives, anti-histamines, common cold tablets and syrups, vitamins and nutritional complements were the commonly used class of medications for self medication purpose [4, 5, 9, 11–14]. Socio-demographic characteristics such as age, gender, participants level of education and monthly income [4, 5] were significantly associated to self medication practice.

In Eritrea, no systematic study has been conducted on self-medication practices among students or the general population. Moreover, there is no guideline or policy for dispensing of medicines that specify which drugs are over the counter or prescription only. Unavailability of guidance on the access to medicine in the country would mean that pharmacies and drug shops could dispense any medicine the customer asked for regardless of a prescription paper. This situation could contribute to the unregulated dispensing of medicines and could encourage self medication to be practiced. Our daily observation also indicate self-medication practice is common among the Eritrean society. People could easily obtain medicines from pharmacies without a prescription or using previous old prescriptions. At times drugs were also available in shops, where people get them any time they want.

In addition, the increasing availability of medicinal products with diversity in its quantities and variety could motivate people to practice self-medication. Therefore, this study was conducted to assess the prevalence of self-medication practices and its influencing factors among students of Asmara College of Health Sciences.

## Methods

### Study design

A descriptive cross sectional study was conducted from 21st May – 23rd June 2018 among the students of Asmara College of Health Sciences(ACHS). The College is located in Asmara the capital city of Eritrea and has a total of 1356 undergraduate (Diploma and Degree Program) and 26 post graduate (Master Program) students studying under four schools namely, School of Allied Health Professions, School of Nursing, School of Pharmacy and School of Public Health.

### Sample size and sampling technique

The sample size for this study was determined by using single population proportion formula (n1 = z2p (1-p)/d2) with the following assumptions z = 1.96 for 95% confidence interval, proportion of self-medication (*p*) = 0.5 (50%) and required margin of errors (d) 0.05. This resulted in an initial sample size of 384.16. Since the total number of students (N) was relatively small (1356), a correction factor was introduced as n2 = (n1*N)/ (N + n1)), which gave a sample size of 299. After adding 5% for non-response, n3 the final sample size was 313. Multistage stratified sampling was used to obtain the samples. First, the college was divided into four strata based on the four schools, then each school was further divided into two strata based on the study programs it has i.e. degree and diploma. The sample size was allocated to each first strata proportionally to its size, then the sample was further divided to the second strata using the same method. Again the second strata were further divided based on the department in each study programs. This gives the last third strata from which the sampling frame was developed for each, except for Public Health in which there was only one stratum since it has a degree program only. Finally, a sample of students was drawn by applying a simple random sampling technique. Regular/upgrading undergraduate students of the college who were in active enrolment during the study period and willing to participate in the study were enrolled.

### Instruments and data collection

A structured closed ended self-administered questionnaire was used to collect the required data. The questionnaire composed of two parts was developed by the authors after a detailed review of the literature. Then it was distributed to experts in the field of pharmacy, public health and research for an expert opinion and was pretested to make necessary amendments. The first section included questions related to the demographic characteristics of participants such as age, gender, study year and study program, average monthly allowance/income, mother’s and father’s education and work. The second section included questions related to the practices, frequency, and duration of self-medication practice, name of the medications used, an ill condition related to the practice, reasons for self-medication practices, source of medication and information, dosage changes, and outcome of the last self-treatment. The selected participants were given group orientation with regard to the aim of the study and introduced with the questionnaire. Finally, completed questionnaires were collected.

### Data entry and analysis

Data was checked for completeness and cleaned manually, and finally, it was coded and entered into SPSS version 23. Descriptive statistics like simple frequencies, mean, median and standard deviation were used to describe the data and chi-square test was used to assess the relationship between the variables. The variables found to have significant × 2 results were used in inferential statistics by using logistic regression, to identify the association between the predictor variables (socio-demographic and other participant’s characteristics) and the outcome variable (self-medication practice). In this study *p* ≤ 0.05 was considered as statistically significant.

## Results

### Socio demographic characteristics

A total of 313 questionnaires were distributed to assess the self-medication practice in which 293 were completed and returned making a response rate of 93.6%. The median age of respondents was 20 and IQR [19–21]. There was almost an equal number of male 50.9%(*n* = 149) and female 48.5% (*n* = 142) participants. Three quarter (75.8%) of the participants were permanent residents of urban areas and majority (87.0%) represented Christian religion. The students were from four schools namely school of Nursing, Allied Health Professions, Pharmacy and Public Health in which 58.7% (*n* = 172) were diploma and 41.3% (*n* = 121) degree program level (Table 1).

**Table 1:** Socio demographic characteristics of respondents

Variable	Self-medication (*n* = 232)	No self-medication (*n* = 61)	Total (%)	*p*-values
Sex (*n* = 291)				
Male	108(72.5%)	41(27.5%)	149 (51.2%)	.005
Female	122 (85.9%)	20 (14.1%)	142 (48.8%)	
Age range				
18–20	138 (75.8%)	44 (24.2%)	182 (62.1%)	.222
21–23	64 (82.1%)	14 (17.9%)	78 (26.6%)	
24–26	11 (91.7%)	1 (8.3%)	12 (4.1%)	
27–54	19 (90.5%)	2 (9.5%)	21 (7.2%)	
Residence (*n* = 289)				
Urban	186 (83.8%)	36 (16.2%)	222 (76.8%)	.000
Rural	42 (62.7%)	25 (37.3%)	67 (23.2%)	
Study program level				
Diploma	131 (76.2%)	41 (23.8%)	172 (58.7%)	.129
Degree	101 (83.5%)	20 (16.5%)	121 (41.3%)	
Average monthly income/allowance(*n* = 287)				
Don’t have income	160 (76.2%)	50 (23.8%)	210 (73.2%)	.012
Have income	69 (95.8%)	8 (4.2%)	77 (26.8%)	
Mother’s education(*n* = 290)				
Illiterate	26 (68.4%)	12 (31.6%)	38 (13.1%)	.041
Can read and write – Elementary	73 (76.0%)	23 (24.0%)	96 (33.1%)	
Middle - Secondary	91 (82.7%)	19 (17.3%)	110 (37.9%)	
Tertiary	36 (90.0%)	4 (10.0%)	40 (13.8%)	
Don’t know	3 (50.0%)	3 (50.0%)	6 (2.1%)	
Father’s education(n = 287)				
Illiterate	9 (69.2%)	4 (30.8%)	13 (4.5%)	.015
Can read and Write - Elementary	47 (75.8%)	15 (24.2%)	62 (25.1%)	
Middle - Secondary	79 (77.5%)	23 (22.5%)	102 (35.5%)	
Tertiary	79 (89.8%)	9 (10.2%)	88 (30.7%)	
Don’t know	11 (91.7%)	1 (8.3%)	12 (4.2%)	

### Self medication practices and prevalence

Overall, 79.2% of the respondents reported they had practiced self medication and 73.3% of the practice happened 6 months prior to the study. Among the participants, 85.9% of females and 72.5% of males practiced self medication. Headache and fever was the major (62.9%) complaint related to self-medication practice followed by common cold 25.0%(*n* = 58), pain and chills 22.4% (*n* = 52), and sore throat 10.3% (*n* = 24). The most frequently appeared reasons that lead participants to such practice were previous experience 51.7% (*n* = 120), perceived sufficient knowledge about drug 35.8%(*n* = 83), perceived mildness of the illness 25.4%(*n* = 59), availability of drugs 24.1%(*n* = 56) and saving time and money 14.7%(*n* = 34) and 7.8%(*n* = 18) respectively (Table 2).

**Table 2:** Self-medication practices and prevalence

Variable	Frequency	Percentage(%)
Ever practiced self-medication (*n* = 293)		
Yes	232	79.2
No	61	20.8
Complaints for self medication		
Headache and fever	146	62.9
Common cold/cough	58	25.0
Pain and chills	52	22.4
Sore throat	24	10.3
Diarrhoea	19	8.2
Vomiting	9	3.9
Others	7	2.8
Skin infection/diseases	6	2.6
Reasons for self medication		
Previous experience	120	51.7
Sufficient knowledge about drugs	83	35.8
Mildness of the illness	59	25.4
Availability of drugs easily	56	24.1
To save time	34	14.7
To save money	18	7.8

The most commonly used classes of medicine for self-medication purpose were analgesics 64.6%, antipyretics 40.7% and anti-bacterial 25.4%. while Ophthalmic, Laxatives, and Cathartic medicines have been less commonly used (Table 3).

**Table 3:** Class of medicines used in self medication

List of medicine used in self-medication	Frequency	Percent(%)
Analgesics	305	64.6
Antipyretics	192	40.7
Antibacterial	120	25.4
Vitamins and minerals	10	2.1
Antiemetic	8	1.7
Anti-acid and anti-ulcer	7	1.5
Anti-fungal	6	1.3
Anti-allergy and medicine used in anaphylaxis	6	1.3
Anti-malarial	5	1.1
Ophthalmic medicine	2	0.4
Laxatives	1	0.2
Cathartic	1	0.2

Among the sources of information about the medicines used for self-medication practices academic knowledge was reported by more than half (51.7%) of the respondents followed by family 29.3%, reading materials 27.3%, previous prescription for similar disease 26.7%, friends/classmate 10.3%, while internet/advertising was insignificant (2.6%). Similarly, pharmacy/drug shop represented the highest Fig. 69.3% as a source of medicine during self medication (Table 3).

Significant number (69.6%) of respondents stated that they did not change the doses during the course of self-treatment, in contrast, 25.2% of respondents changed the dose at times and 5.2% changed it every time during the self-medication practices. Among the reasons that lead to dosage change, worsening illness was mentioned by 30.0% of the students, fear of side effect and dosage insufficient for the condition each accounted 24.3% and improving illness was mentioned by 21.4% of the students. Adverse drug reactions were also reported by 9.2% (*n* = 21) respondents. Regarding the attitude to self-medication practice, half (55.2%) of subjects thought self-medication is effective but only 35.9% recommends self-medication practice to others (Table 4).

**Table 4:** Factor associated with self-medication practices

Variable	Frequency	Percent(%)
Drug information source		
Academic knowledge	120	51.7
Family	68	29.3
Reading materials	63	27.2
Previous prescription for similar diseases	62	26.7
Friend/class mate	24	10.3
Internet/Advertising	6	2.6
Source of drug for Self medication		
Pharmacy/drug shop	160	69.3
Family/relative	62	26.8
Left over from previous prescription	31	13.4
Friend/class/roommate	21	9.1
Have you ever faced with adverse drugs reaction from self-medication practice(*n* = 228)		
No	188	82.5
Yes	21	9.2
Not certain	19	8.3
Do you recommend or advice self-medication to others (*n* = 284)		
No	127	44.7
Yes	102	35.9
Not certain	55	19.4

### Determinants of self medication practice

In calculating the logistic regression, self medication was the dependent variable and socio demographic characteristics were independent variables. The results showed that females were 2.8 times more likely to self-medicate themselves (AOR: 2.84[CI: 1.33–6.05], *p* < .01) than males. Those who were permanent residents of urban areas tend to practice self-medication more often (COR: 3.07 [CI: 1.67–5.66], *p* < .001) than those who live in rural. Based on monthly income or allowance, the respondents were categorized into two groups, and those who reported to have monthly allowance were found to be 5.5 times more likely (AOR: 5.53 [CI: 1.96–15.63], *p* < .01) to practice self-medication than those who do not have.

Based on year of study and the school in which they enrol, third year students were found to practice self-medication more often (COR: 2.73 [CI:1.16–6.43], *p* < .05) than the others, and students of Allied Health Professions (AOR: 7.82 [CI: 2.08–29.44], *p* < .01), Pharmacy (AOR: 25.75 [CI: 4.85–136.70], *p* < .001), and Nursing (AOR: 6.19[CI: 1.77–21.59], *p* < .01) had higher odds (7.82, 25.72, 6.19 respectively) of self medication practice than the students of Public Health. Mother’s education was one of the variables assumed to influence self medication practice and logistic regression showed that students who had mothers with tertiary level of education were more likely (COR: 4.154[CI: 1.203–14.339], *p* < .05) to report self medication. In addition, students whose mothers had any kind of job had 2.56 higher odds (COR: 2.56 [CI: 1.15–5.68], p < .05) to use self medication as compared to students whose mothers were housewives. Fathers’ work was categorized into two groups, group one included those who are farmers or are currently in military and group two included those who are either government workers(civil) or engaged in other private businesses. Accordingly, students whose fathers’ work lay in group two had an odds of self-medication that increased 2.1 fold as compared (COR: 2.14[CI: 1.20–3.81], *p* < .05) to students whose fathers are either farmers or in the military (Table 5).

**Table 5:** Logistic regression analysis

Variable	Self-medication (*n* = 232)	No self-medication (*n* = 61)	COR[95% CI]	AOR[95% CI]
Sex (*n* = 291)				
Male	108(72.5%)	41(27.5%)		
Female	122 (85.9%)	20 (14.1%)	2.31 [1.27–4.19]**	2.84[1.33–6.05]**
Residence (*n* = 289)				
Rural	42 (62.7%)	25 (37.3%)		
Urban	186 (83.8%)	36 (16.2%)	3.07 [1.67–5.66]***	
Monthly income/ allowance(*n* = 287)				
Don’t have income	160 (76.2%)	50 (23.8%)		
Have income	69 (95.8%)	8 (4.2%)	2.69 [1.21–5.98]*	5.53[1.96–15.63]**
Year of study				
First year	78 (73.8%)	28 (26.4%)		
Second year	56 (73.7%)	20 (26.3%)		
Third year	61 (88.4%)	8 (11.6%)	2.73[1.16–6.43]*	
Fourth year	37 (88.1%)	5 (11.9%)		
School				
Public Health	15 (62.5%)	9 (37.5%)		
Allied Health Professions	72 (80.0%)	18 (20.0%)		7.82[2.08–29.44]**
Nursing	84 (75.0%)	28 (25.0%)		6.19 [1.77–21.59]**
Pharmacy	61 (91.0%)	6 (19.0%)	6.1[1.87–19.79]**	25.75 [4.85–136.70]***
Mother’s education(n = 290)				
Illiterate	26 (68.4%)	12 (31.6%)		
Can read and write – Elementary	73 (76.0%)	23 (24.0%)	1.465 [.639–3.357]	
Middle - Secondary	91 (82.7%)	19 (17.3%)	2.211 [.951–5.141]	
Tertiary	36 (90.0%)	4 (10.0%)	4.154 [1.203–14.339]*	
Mother’s work(*n* = 290)				
Don’t have work	65 (89.0%)	8 (11.0%)		
Have work	165 (76.0%)	52 (24.0%)	2.56 [1.15–5.68]*	
Father’s work(*n* = 284)				
Group 1	78 (70.9%)	32 (29.1%)		
Group 2	146 (83.9%)	28 (16.1%)	2.14[1.20–3.81]*	
N.B. p ≤ 0.05(*), *p* ≤ 0.01(**), *p* ≤ 0.001(***)				

## Discussion

Several studies revealed that self-medication practice is common and the prevalence varies throughout the world. The prevalence of self-medication practice in this study was 79.2%. Similar prevalence has been reported in studies conducted among university students from Serbia 79.9% [13], India 78.6% [9], Nepal 81.9% [14], Egypt 62.9% [10] and South-western Nigeria 91.4% [4], but two studies from Ethiopia 32.7% [5] and 38.5% [11] and another study from Iran 33.7% [12] reported lower prevalence. The studies from Serbia, India, Iran, Nepal, and Ethiopia (11) were conducted among medical students. While the study from Egypt was conducted among medical and non-medical and the study from Ethiopia (5) was among social science students. High prevalence of self-medication practices among the students of ACHS could be due to higher knowledge, education in health sciences and their clinical exposures. In addition, the difference in self-medication practice among countries could be attributed to the difference in socioeconomic and demographic characteristics of participants.

Results of this study showed that the first main complaint related to self medication was headache and fever 62.9% followed by common cold 25.0%, pain and chills 22.4%. Some studies reported comparable observations in which fever and headache were the leading complaints [9, 11, 14]. A study in Egypt found cold, headache, sore throat, intestinal colic, and cramps among the frequent complaints that encouraged self-medication practice [10]. Whereas a study in south western Nigeria reported urinary tract infection, sore throat and diarrhoea as the main complaint or diseases related to self-medication practices [4].

In the present study, the top three reasons that lead participants to self medication practice were a previous experience of treating the same ill condition 51.7%, perceived sufficient knowledge about drug 35.8% and perceived mildness of the illness 25.4%. This complies with the results of former studies that described a previous experience as the first main reason [11, 15, 16] but, contrary to studies that reported symptoms were not serious or mildness of the disease as a leading reason [5, 9, 13]. However, practicing self medication based on previous experience of treating the same ill condition couldn’t be a guarantee as there could be a misdiagnosis of the earlier or current ill condition which could lead to incorrect treatment choice and subsequent unwanted health problems.

The recurrently used classes of medicine for self-medication purposes in this study were analgesics 64.6%, antipyretic 40.7% and anti-bacterial 25.4%. Similar findings were observed in a study from Nepal in which painkillers, anti-pyretic and antimicrobials were among the drugs commonly used for self-medication practice [14]. A number of studies reported that at least one from analgesics, or anti-pyretic or anti-biotic were the first three frequently used medicines [5, 9, 11–13]. In Nigeria, anti-malarial was the first most commonly reported class of medicines for self medication but in our case, it was found to be among the least reported medicines which could be due to the difference in study setting as the study in Nigeria was conducted in malaria endemic area [4]. Generally, the reason behind the selection of these medicines could be due to the confidence of the subjects in identifying the signs and symptoms of a particular disease they faced which was evidenced by the high proportion of participants perceiving having sufficient knowledge and or experience with drugs or it could be due to the easy accessibility of these medications.

Some previous literature testified pharmacy or drug shop as the main source of medication and friends, relatives and left over from previous prescription represented some of the frequently reported sources [4, 10–12]. The current study also had a similar finding with pharmacy/drug shop accounting 69.3% as the main source. Easy accessibility of all medications (even those medicine which should only be dispensed with physicians’ prescription) from pharmacies and drug shops could be related to the to the absence of legislation regarding access to medicine in our country. This legislation gap could contribute to an increasing number of individuals who could practice self medication. Thus, leading to irrational drug use and potential development of drug resistance and likely harm on human life. Furthermore, the study revealed that for more than half (51.7%) of the respondents’, academic knowledge was the primary sources of medicine information with family 29.3%, reading materials 27.3%, and a previous prescription for similar disease 26.7% among the sources in the list. Internet the second most common source of information in Iran [12] was the least (2.6%) reported source in our case. This was an expected result as the internet service in our country is limited to some urban areas and its quality is very poor. Other studies reported different top drug information sources such as reading material, an old prescription for the same disease and pharmacy clerk [9–11].

Changes in the dosages of medication during the course of self-medication was reported by 30.4% of the respondents of the current study and higher (57.1%) percentage of dosage change had been reported in another studies [17]. Adverse drug reaction following the use of self medication practice was reported by 9.2% of the subjects which is nearly double of the previous study finding that quantified 5% experience of adverse drug reaction [18]. In the current study, more than half (55.2%) of subjects had a positive attitude about self-medication practice and 35.9% of respondents suggested the practice to others. A similar finding was observed in former studies with 55.5% of the participants agreed on the practice of self-medication [11], at the same time 64% [18] and (68.2%)of respondents advised medication to others [19].

In this study, results of logistic regression showed that females were 2.8 times more likely to self-medicate themselves (AOR: 2.84[CI: 1.33–6.05], *p* < .01) than males which is parallel to previous studies finding [5, 9, 10, 12, 15]. In Nigeria, self-medication was higher in females than in males and there was a significant association with age, gender, and participants level of education [4]. In Serbia, female respondents practiced self-medication 1.4 times more often than male respondents [13]. In contrary to this, some studies reported that there was no significant difference in self-medication practice between males and females [11, 16]. This gender difference in self-medication practice could be explained by the special health conditions such as menstruation that women face which could be obvious complaint with clear measures to take from their point of view.

Similarly, participants whose permanent residence was in urban areas tend to practice self-medication more often (COR: 3.07 [CI: 1.67–5.66], *p* < .001) than those who live in rural areas. An earlier study from Egypt came with a result that showed being from urban areas was an independent predictor for self medication [10]. The reason for such variation of the practice with residence could be associated with the difference in accessibility to health care service. Based on monthly income or allowance, those who reported to have monthly allowance were found to be 5.5 times more likely, (AOR: 5.53 [CI: 1.96–15.63], *p* < .01) to practice self-medication than those who did not have. Results from a study conducted in Ethiopia were in agreement with this finding that high monthly income was shown to encourage self medication [5]. The reason for such finding could be described by the fact that, in our setting students are eligible for free medication from government owned hospitals or pharmacies if they present a physician’s prescription along with the fee exemption paper (sick report), otherwise students have to buy from private pharmacies. Therefore, students with some monthly allowance have the chance of buying medicines directly rather than visiting hospitals to obtain the prescription and fee exemption paper. In this study, third year students were found to practice self-medication 2.7 times more often (COR: 2.73 [CI:1.16–6.43], *p* < .05) than the others. Similar observations were reported from Southwest Nigeria and Ethiopia that stated a significant association between study years and self medication [4, 13]. But, another study from Ethiopia and Peru found no statistically significant association between years of study [5, 20]. A study from Mekele University in Ethiopia reported a significant difference between departments, pharmacy students practiced self-medication more than medical and paramedical students [15]. In the present study, students of Allied Health Professions (AOR: 7.82 [CI: 2.08–29.44], *p* < .01), Pharmacy (AOR: 25.75 [CI: 4.85–136.70], *p* < .001), and Nursing (AOR: 6.19[CI: 1.77–21.59], p < .01) had higher odds of self-medication practice than the students of Public Health. This could be due to the nature of the curriculum, as the students of public health spend most of their practical session in more of community setting whereas the others were directly involved with patient care (clinical area) which could have increased both the accessibility and knowledge of medications.

Univariate logistic regression showed that students who had mothers with tertiary level of education were more likely (COR: 4.154[CI: 1.203–14.339], *p* < .05) to report self medication practice. In a study conducted in Serbia, a high level of mothers’ education was independently associated with self medication practice of prescription only drugs. In addition, students whose mothers had any kind of job were more likely (COR: 2.56 [CI: 1.15–5.68], *p* < .05) to use self medication as compared to students whose mothers were housewives. This could be due explained as working mothers could have less time but more income as compared to a housewife. Thus, they could practice self-medication which may in turn, encourage their children to practice self-medication. Students whose fathers’ work in government offices or private offices were 2.14 times more likely (COR: 2.14[CI: 1.20–3.81], p < .05) to use self medication as compared to students whose fathers are either farmers or in the military. The differences in self medication practice between these two groups could be attributed to the difference in income, accesses to medication and information these two groups had.

## Conclusion

High prevalence of self-medication practice has been observed among students of Asmara College of Health Sciences. In this study, sex, income, and department of study were found to be the independent predictors for self-medication practice. Therefore, students should be educated on the consequences of self-medication practices. Moreover, national guideline on medicine access should be developed and strong measures should be implemented to halt the selling of medications without a proper prescription. Further studies should also be done to assess the practice in community.

### Limitation of the study

This study was done among health science students and comparison group from different streams (non-health sciences) is lacking. Recall bias could have happened with some question and as the questionnaire was self-administered respondents could have discussed among them.

## Abbreviations

ACHS: Asmara College of Health Sciences
